# GLP-1 receptor agonists and lymphedema: from weight loss to potential direct modulation of lymphatic function

**DOI:** 10.3389/fendo.2026.1980239

**Published:** 2026-10-08

**Authors:** Eva Klara Merzel Šabović, Miodrag Janić, Tanja Planinšek Ručigaj

**Affiliations:** 1 Clinic of Dermatovenereology, University Medical Centre Ljubljana, Gradiškova, Ljubljana, Slovenia; 2 University of Ljubljana, Faculty of Medicine, Vrazov trg, Ljubljana, Slovenia; 3 Department of Endocrinology, Diabetes and Metabolic Diseases, University Medical Centre Ljubljana, Zaloška, Ljubljana, Slovenia

**Keywords:** disease modification, GLP-1 receptor agonists, inflammation, lymphatic function, lymphedema, obesity, semaglutide

## Abstract

Obesity and lymphedema are linked through increased interstitial fluid load, impaired collecting-vessel pumping, inflammation, and progressive fibro-adipose remodeling. This bidirectional relationship makes glucagon-like peptide-1 receptor agonists (GLP-1RAs) relevant through two potentially distinct pathways. First, substantial weight loss may unload a compromised lymphatic system, improve mobility, and reduce adipose-driven inflammation. Second, GLP-1 signaling may directly influence lymphatic vessel function. Case reports and a retrospective cohort have described reduced limb volume, improved symptoms or imaging findings, and a lower incidence of breast cancer-related lymphedema among GLP-1RA users. Experimental work has identified GLP-1 receptors in lymphatic endothelial cells and shown that pharmacological activation with semaglutide increases the pumping capacity of isolated collecting lymphatic vessels. These findings support a potential direct lymphatic effect but do not establish clinical efficacy in human lymphedema. Improvement during treatment may instead reflect reduced fat mass, greater mobility, or changes in conventional care. Future studies should distinguish weight-loss-dependent unloading from weight-loss-independent effects by combining standardized clinical outcomes, functional imaging, body-composition measurements, and, where feasible, weight-matched comparison groups. The available evidence therefore supports prospective investigation rather than clinical adoption. Trials should assess early vascular responses before major weight loss, longer-term changes in tissue composition and inflammation, and durability after treatment withdrawal, while maintaining standardized compression, exercise, skin care, and other components of established lymphedema management throughout. Until such evidence is available, GLP-1RAs should be prescribed for established metabolic indications rather than lymphedema itself, and any lymphatic benefit should be regarded as a promising clinical signal rather than a treatment claim.

## 1 Introduction

Lymphedema begins with inadequate lymphatic transport, but chronic disease is no longer simply a problem of retained fluid. Protein-rich stasis reshapes the affected tissue. Adipocytes accumulate, immune-cell traffic changes, and collagen deposition progressively reduces tissue compliance (1, 2). The clinical manifestations of chronic lymphedema therefore reflect both impaired lymphatic transport and the subsequent tissue response.

Obesity may contribute to lymphatic dysfunction through multiple, potentially reinforcing mechanisms. It increases the amount of fluid that must be cleared and, at the same time, weakens the system responsible for clearing it. Poor lymphatic transport may then encourage regional fat deposition and inflammation. Experimental and clinical observations support this bidirectional relationship, although its relative importance is likely to vary between obesity-induced, cancer-related, and primary lymphedema (3–9). That feedback loop creates a therapeutic opening. Successful obesity treatment could reduce lymphatic load, improve mobility, and dampen adipose-driven inflammation. Glucagon-like peptide-1 receptor agonists (GLP-1RAs) are particularly interesting because the weight loss achieved with these drugs is larger and more sustained than that usually obtained with lifestyle measures alone. The recent demonstration of GLP-1 receptors in lymphatic endothelium raises a further question: could these agents also act on the lymphatic vasculature itself (10)?

The purpose of this Perspective is to define that question carefully. Weight reduction is an established effect of GLP-1RA therapy; whereas direct modulation of lymphatic function remains unproven. Distinguishing between these two mechanisms is important to avoid overstating the limited clinical evidence and, more usefully, to identify experiments that could differentiate one mechanism from the other.

## 2 The obesity-lymphatic dysfunction cycle

Body size is only part of the link between obesity and lymphatic failure. As adiposity increases, capillary filtration and interstitial fluid formation rise. Reduced movement, loss of the skeletal-muscle pump, venous hypertension, and mechanical constraint of superficial lymphatics further narrow the available transport reserve. Clinical swelling appears when that reserve is no longer sufficient (3–6).

The relationship between obesity and lymphatic dysfunction manifests differently depending on the specific adipose tissue depot. In obesity, subcutaneous adipose tissue (SAT) exhibits a regression of lymphatic capillaries and decreased lymphatic density. While collecting lymphatic vessels remain and may enlarge, the surrounding lymphatic muscle cells (LMCs) lose their intrinsic rhythmic pacemaker activity and mechanical responsiveness, leading to a net reduction in vessel contraction and lymphatic drainage. Visceral adipose tissue (VAT), on the other hand, naturally contains approximately fivefold more lymphatic vessels than SAT. In obesity, VAT undergoes excessive and disordered lymphangiogenesis, largely driven by profound, roughly 100-fold increases in vascular endothelial growth factor C (VEGFC). This results in dilated, highly permeable collecting vessels with shortened valves that often remain open, disrupting unidirectional flow and facilitating retrograde lymph leakage (11).

Adipose tissue also changes the local biology. Hypertrophic fat becomes relatively hypoxic and attracts macrophages and other immune cells. These inflammatory and molecular drivers profoundly impact lymphatic function. Obesity drives the accumulation of perivascular macrophages and T cells around collecting lymphatic vessels, which upregulate inducible nitric oxide synthase (iNOS). The resulting rise in NO profoundly dilates the lymphatic vessels and directly inhibits their intrinsic pumping ability. Increased levels of tumour necrosis factor (TNF) in obese adipose tissue directly weaken the barrier integrity of lymphatic endothelial cells, promoting lymph extravasation into the surrounding tissue. TNF also hyperpolarizes LMCs, which actively suppresses their contraction frequency (11). Altered adipokine signaling, oxidative stress, and the localized accumulation of free fatty acids from basal lipolysis further disturb lymphatic endothelial integrity. Furthermore, localized insulin resistance within the lymphatic endothelial and muscle cells may diminish their identity, viability, and contractility (3–5, 7, 8, 11).

Once stasis is established, the affected tissue becomes part of the disease process. Persistent immune activation promotes fibroblast activity, extracellular-matrix deposition, and adipocyte expansion. Fibrosis reduces tissue compliance and may further constrain lymphatic channels; accumulated fat provides an additional inflammatory stimulus. Thus, obesity can impair lymphatic transport, while impaired transport can promote the very fat deposition and fibrosis that perpetuate it (1, 2, 8–10).

Not every patient will therefore respond in the same way. Early disease may retain a sizeable functional component, whereas advanced fibro-adipose remodeling may be only partly reversible (6, 8, 10). Disease duration, recurrent cellulitis, prior nodal surgery or radiotherapy, and the extent of fibrosis may matter as much as baseline body mass index.

Representative clinical and experimental studies are summarized in **Table 1**, and the bidirectional relationship between obesity and lymphatic dysfunction is illustrated in **Figure 1**.

**Table 1:** Representative clinical and experimental studies examining the relationship between obesity and lymphedema.

Study	Design and population	Main findings	Interpretation/limitations
Helyer et al., 2010 (23)	Prospective measurements; 137 women after breast cancer surgery; ≤24 months	Lymphedema: 11.6%. BMI >30 vs <25 kg/m²: OR 2.93 (95% CI 1.03–8.31)	Obesity predicted BCRL; few events and a relatively small cohort
Fu et al., 2015 (24)	Prospective cohort; 140 women; 12 months after breast cancer surgery	Obese women more often had abnormal bioimpedance before surgery and at 4–8 weeks; similar trend at 12 months	Possible pre-existing lymph-fluid disturbance; bioimpedance outcome was not clinical lymphedema
Greene et al., 2015 (6)	Clinical/lymphoscintigraphic study; 51 patients with BMI >30 kg/m² and no other cause of leg lymphedema	Abnormal vs normal lymphatic function: maximum BMI 72.0 vs 37.7 kg/m² (p<0.0001). Findings were abnormal at BMI >60 and normal at <50	Extreme obesity may directly cause lymphatic failure; selected cohort and nonabsolute BMI thresholds
Burian et al., 2024 (LIMPRINT) (25)	International cross-sectional study; 7,397 patients; 38 centers in nine countries	ISL stage III: 14% without obesity, 18% with class I–II obesity, and 39% with class III obesity; obesity also accompanied bilateral disease, cellulitis, and poorer control	Large real-world study linking obesity with severity; cross-sectional design precludes causality
Shaw et al., 2007 (26)	Three-arm randomized trial; 64 women with BCRL; 24 weeks	Dietary groups lost weight. Excess arm-volume reduction was not significant between groups but correlated with weight loss (r=0.423; p=0.002)	Possible benefit, but small sample and correlation-based result
Schmitz et al., 2019 (WISER) (27)	Randomized trial; 351 overweight BCRL survivors; exercise, weight loss, combined treatment, or control; 12 months	Weight-loss groups lost approximately 7–8%, without significant improvement in BCRL outcomes	Weight loss did not measurably improve established BCRL over one year
Roberts et al., 2021 (28)	Prospective surveillance; 1,161 women after unilateral breast surgery; mean follow-up 49.1 months	BCRL developed in 7.9%. Net weight loss did not reduce risk vs weight gain: HR 1.38 (95% CI 0.89–2.13)	Weight loss did not prevent BCRL; weight change was not randomized
Harvie et al., 2024 (BE-WEL) (29)	Feasibility randomized trial; 57 women with overweight/obesity and BCRL; 12 weeks	Modest weight loss and numerical reductions in relative arm volume; estimates were imprecise and mostly included no effect	Feasible intervention; insufficient power for efficacy conclusions
Blum et al., 2014 (5)	High-fat-diet mouse model	Obesity reduced collecting-vessel contraction frequency and mechanostimulation response; function declined with body weight	Mechanistic evidence for direct impairment; uncertain human translation
Garcia Nores et al., 2016 (30)	Diet-induced obesity in mice	Perilymphatic inflammation and iNOS increased; T-cell or iNOS inhibition improved pumping and fluid clearance	Inflammation may mediate obesity-related lymphatic dysfunction
Torrisi et al., 2016 (31)	Diet-induced obesity in mice	Perivascular macrophages and T cells upregulate iNOS; the resulting elevation in nitric oxide dilates vessels and heavily impairs lymphatic pumping.	Identifies macrophage and T cell-derived nitric oxide as a direct molecular driver of lymphatic contractile failure.
Castorena-Gonzalez, 2022 (32)	Western diet-fed mice	Western diets result in shortened mesenteric collecting lymphatic valves that remain open, disrupting flow and facilitating retrograde leakage.	Demonstrates that diet composition (Western vs. standard high-fat) structurally disrupts unidirectional flow.
Nitschké et al., 2017 (33)	Inducible transgenic mouse model	Adipose-specific overexpression of VEGFC in visceral fat drives excessive, disordered lymphangiogenesis, resulting in dilated, leaky vessels and fatal chylothorax.	Explains how obesity-induced spikes in visceral VEGFC can severely impair, rather than rescue, lymphatic function.
Nitti et al., 2016 (8)	Diet-induced obesity followed by weight loss in mice	Function declined and inflammation increased with weight. >25% weight loss normalized pumping, density, permeability, and clearance	Reversibility demonstrated in mice, but not consistently in human trials
Chakraborty et al., 2019 (34)	Adipose-specific VEGF-D overexpression in obese mice	Lymphatic expansion improved glucose clearance and reduced insulin, liver triglycerides, and adipose macrophages	Supports bidirectional lymphatic–metabolic interactions

BCRL, breast cancer-related lymphedema; BMI, body mass index; CI, confidence interval; HR, hazard ratio; iNOS, inducible nitric oxide synthase; ISL, International Society of Lymphology; OR, odds ratio; VEGF-D, vascular endothelial growth factor D. VEGFC, vascular endothelial growth factor C.

**Figure 1:**
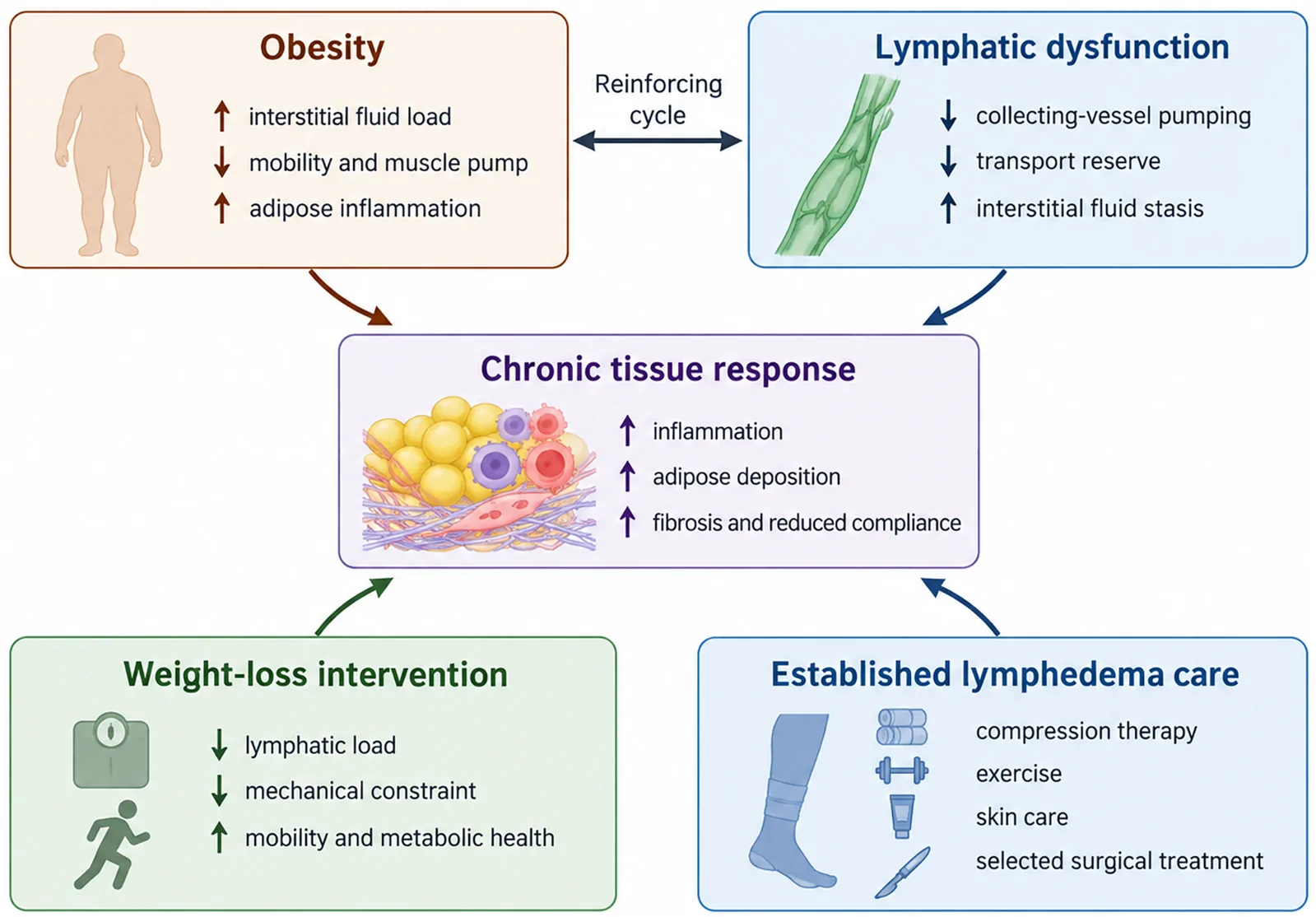
Bidirectional relationship between obesity and lymphatic dysfunction and potential points of intervention.

## 3 What can weight loss reverse?

Weight reduction acts upstream of several stresses at once. It can lower interstitial fluid formation, relieve mechanical constraint, improve mobility, and reduce the inflammatory output of dysfunctional adipose tissue (6–8). From a mechanistic standpoint, reducing hypertrophic fat limits the excessive basal lipolysis that releases free fatty acids (such as palmitate and oleate), which directly compromise lymphatic barrier integrity. Furthermore, weight loss has been shown to reduce the accumulation of perivascular macrophages and T cells, thereby lowering localized iNOS activity and removing the continuous suppression of lymphatic vessel contraction (11).

These effects are clinically relevant even if the lymphatic vasculature itself does not recover completely. A smaller limb, however, does not by itself demonstrate restored lymphatic function. Circumference may fall because regional fat mass decreases, extracellular fluid shifts, or overall mobility improves. These are worthwhile outcomes, but they answer a different question from whether intrinsic lymphatic vessel pumping and barrier integrity have actually been repaired (11).

Timing and tissue pathology dictate what is recoverable. Preclinical models demonstrate that substantial weight loss (e.g., >25%) can normalize lymphatic pumping, permeability, and tracer clearance when the abnormality is still predominantly functional. However, once advanced fibro-adipose remodeling is established, such as severe extracellular matrix deposition, permanent valve atrophy, or irreversible structural disruption of the lymphatic channels, complete normalization is far less likely. Future trials should therefore distinguish generic symptomatic benefit from true functional recovery, and not define disease modification on the basis of limb volume alone (6, 8, 11, 12).

## 4 Why GLP-1 receptor agonists may be different

GLP-1RAs produce weight loss large enough to alter the mechanical and metabolic setting in which lymphedema develops (13, 14). For people living with obesity, this is the most secure basis for expecting benefit: less adipose tissue, lower lymphatic load, improved insulin sensitivity, and often greater mobility. The pathway is unlikely to be purely mechanical. Loss of visceral and ectopic fat changes adipokine balance and systemic inflammation, while improved mobility strengthens muscle-assisted fluid clearance. Better metabolic health may also make compression, exercise, and skin care easier to sustain. None of this, however, requires a direct action on a lymphatic vessel.

The case for a potential direct action is newer and is uniquely supported by the natural physiology and bioavailability of GLP-1 within the lymphatic system. Endogenous GLP-1 is secreted from intestinal L cells directly into the mesenteric lymph, reaching concentrations up to 80-fold higher than those found in the bloodstream. This high lymphatic exposure is facilitated by substantially lower levels of the GLP-1-degrading enzyme dypeptidil peptidase-4 (DPP-4) in the lymph compared to the blood. GLP-1 receptor expression is enriched in collecting lymphatic vessels and potentially on lymphatic muscle cells, but these receptors are absent on capillary lymphatic endothelial cells (11). Experimental work by Schulz and colleagues demonstrated that pharmacological activation with semaglutide directly increases the pumping capacity of isolated collecting lymphatic vessels (10). This functional restoration has been observed across multiple models, including vessels from wild-type, diet-induced obese, hypercholesterolemic and Western diet-fed ApoE-knockout mice. Ex vivo assessments reveal that this semaglutide-enhanced lymphatic contraction is partly mediated by nitric oxide (NO) signaling, prostaglandins (as shown by attenuation with COX1 and COX2 inhibitors), and NADPH-mediated reactive oxygen species (ROS); although the complete downstream signaling pathway remains incompletely defined (10, 11).

These findings provide a biologically plausible mechanism for a direct lymphatic effect, but they do not demonstrate that a fibrotic human limb will respond in the same way. Conflating these pathways would weaken the argument. Weight-loss-mediated unloading is plausible and clinically accessible now, a drug-specific effect on lymphatic contractility or repair remains a hypothesis.

The distinction is not semantic: it determines the comparator, the timing of measurements, and what may legitimately be called disease modification. Furthermore, as dual glucose dependent insulinotropic polypeptide (GIP)/GLP-1 RAs (such as tirzepatide) drive even greater weight loss, their specific effects on the lymphatic system warrant future investigation, especially considering that GIP is also found at high levels in the mesenteric lymph. As the landscape of incretin-based pharmacotherapies rapidly evolves, the development of oral small-molecule GLP-1 RAs (such as orforglipron) and novel multi-agonist peptides (including the glucagon/GLP-1 dual agonist survodutide and the glucagon/GIP/GLP-1 triple agonist retatrutide) may offer new therapeutic avenues. Future studies should investigate whether these emerging small molecules or triple-agonists penetrate the lymphatic compartment or relieve lymphatic load more effectively than currently available injectables (15).

## 5 What does the current evidence show?

The human evidence is still sparse. Crowley and colleagues described a woman with breast cancer-related lymphedema whose 24% weight loss during semaglutide therapy was accompanied by a reduction in limb-volume difference from 10.3% to 3.4%, freedom from compression, better patient reported function, and improved indocyanine-green lymphography (16). In a second report, a man with extreme obesity and massive localized lymphedema lost 40 kg over 28 weeks; thigh circumference fell by at least 16 cm and mobility improved (17). Both cases are clinically striking, but neither can isolate a direct lymphatic effect from weight loss, renewed mobility, nutritional treatment, or changes in usual care.

Brown and colleagues subsequently examined 3,830 patients after axillary lymph-node dissection, of whom 76 had received a GLP-1RA. Lymphedema occurred in 6.6% of users and 28.5% of non-users; the adjusted association remained large (18). The result is difficult to dismiss, but it is equally difficult to interpret causally. Exposure was not randomized, the treated group was small, and unmeasured differences in weight trajectory, metabolic care, cancer treatment, behavior, and surveillance may have influenced the finding.

Experimental evidence now provides a more direct biological link. Schulz and colleagues demonstrated GLP-1 receptor expression in lymphatic endothelial cells and showed that semaglutide increased the pumping capacity of isolated collecting lymphatic vessels from wild-type, diet-induced obese, and hypercholesterolemic mice (10). The finding is particularly relevant because the effect was observed at the level of lymphatic vessel function rather than as a secondary consequence of weight loss. However, the experiments were performed in isolated murine vessels, and therefore cannot establish whether the same mechanism operates in patients with chronic human lymphedema, particularly when anatomical disruption, fibrosis, or recurrent inflammation are dominant constraints.

The present evidence therefore occupies an intermediate position: it is stronger than conjecture but well short of therapeutic proof. The ongoing prospective study should refine the clinical signal (10), although its single-arm design will not by itself resolve whether improvement is caused by weight loss or by a drug-specific lymphatic action.

The available clinical and experimental evidence linking GLP-1 receptor agonists with lymphatic outcomes is summarized in **Table 2**.

**Table 2:** Current evidence linking GLP-1 receptor agonists with lymphatic outcomes.

Evidence source	Population or model	Main observation	Interpretation
D’Alessio et al. (2007) (35), Ohlsson et al. (2014) (36)	Human/Experimental physiological models	Endogenous GLP-1 is secreted into mesenteric lymph at concentrations up to 80-fold higher than in blood, aided by significantly lower levels of the degrading enzyme DPP-4.	Establishes high natural lymphatic bioavailability, providing a strong physiological rationale for direct lymphatic targeting.
Crowley et al. (2024) (20)	Case report; breast cancer-related lymphedema	Weight loss accompanied by lower limb-volume differential, symptom improvement, and improved ICG lymphography	Objective signal, but no control and weight loss cannot be separated from a direct drug effect
Nguyen et al. (2024) (21)	Case report; extreme obesity	Marked weight loss with improvement in massive localized lymphedema and mobility	Clinically relevant, but phenotype and concurrent weight loss limit mechanistic inference
Brown et al. (2024) (22)	Patients undergoing axillary lymph-node dissection	GLP-1RA exposure associated with lower incidence of breast cancer-related lymphedema	Supports a clinical signal; non-randomized and susceptible to residual confounding
Schulz et al. (2026) (10)	Human/experimental lymphatic endothelium and isolated murine collecting lymphatic vessels	GLP-1 receptors were enriched in lymphatic endothelial cells; semaglutide increased pumping capacity of isolated collecting lymphatic vessels, including vessels from diet-induced obese and hypercholesterolaemic mice	Provides direct experimental support for a potential GLP-1-mediated effect on lymphatic vessel function, but does not establish efficacy in chronic human lymphedema
NCT07012642 (37)	Prospective single-arm clinical study	Evaluates semaglutide-associated change in breast cancer-related lymphedema	Useful prospective evidence expected; design will not fully separate weight loss from drug-specific effects

DPP-4, dipeptidyl peptidase-4; GLP-1, glucagon-like peptide-1; ICG, indocyanine green; GLP-1RA, glucagon-like peptide-1 receptor agonist.

The table deliberately separates clinical observations from mechanistic evidence; none of the available studies establishes clinical disease modification.

## 6 A testable disease-modification hypothesis

A useful working model to evaluate the impact of GLP-1RAs on lymphedema relies on two distinct, though potentially coexisting, routes of action: systemic metabolic unloading and direct lymphatic modulation. In the first, the-weight-loss-dependent pathway, the primary driver of lymphatic recovery is the substantial reduction in adipose tissue mass. Weight loss relieves mechanical constraints on superficial lymphatics, lowers the interstitial fluid load, and dampens the systemic output of adipose-derived inflammatory mediators. Furthermore, the loss of hypertrophic fat reduces basal lipolysis and the localized accumulation of free fatty acids that actively paralyze LMCs. In the second, direct targeted lymphatic modulation, GLP-1 receptor activation acts directly on lymphatic endothelium to counteract obesity-induced functional decline. Because endogenous GLP-1 natively circulates at high concentrations within the lymphatic system, pharmacological GLP-1RA therapy may directly stimulate GLP-1 receptors localized on collecting-vessel LMCs. This targeted activation is hypothesized to restore intrinsic pacemaker activity by dampening iNOS/nitric oxide-mediated hyperpolarization, countering TNF-induced barrier permeability, and mitigating ROS stress (11). This two-pathway hypothesis is illustrated in **Figure 2**.

**Figure 2:**
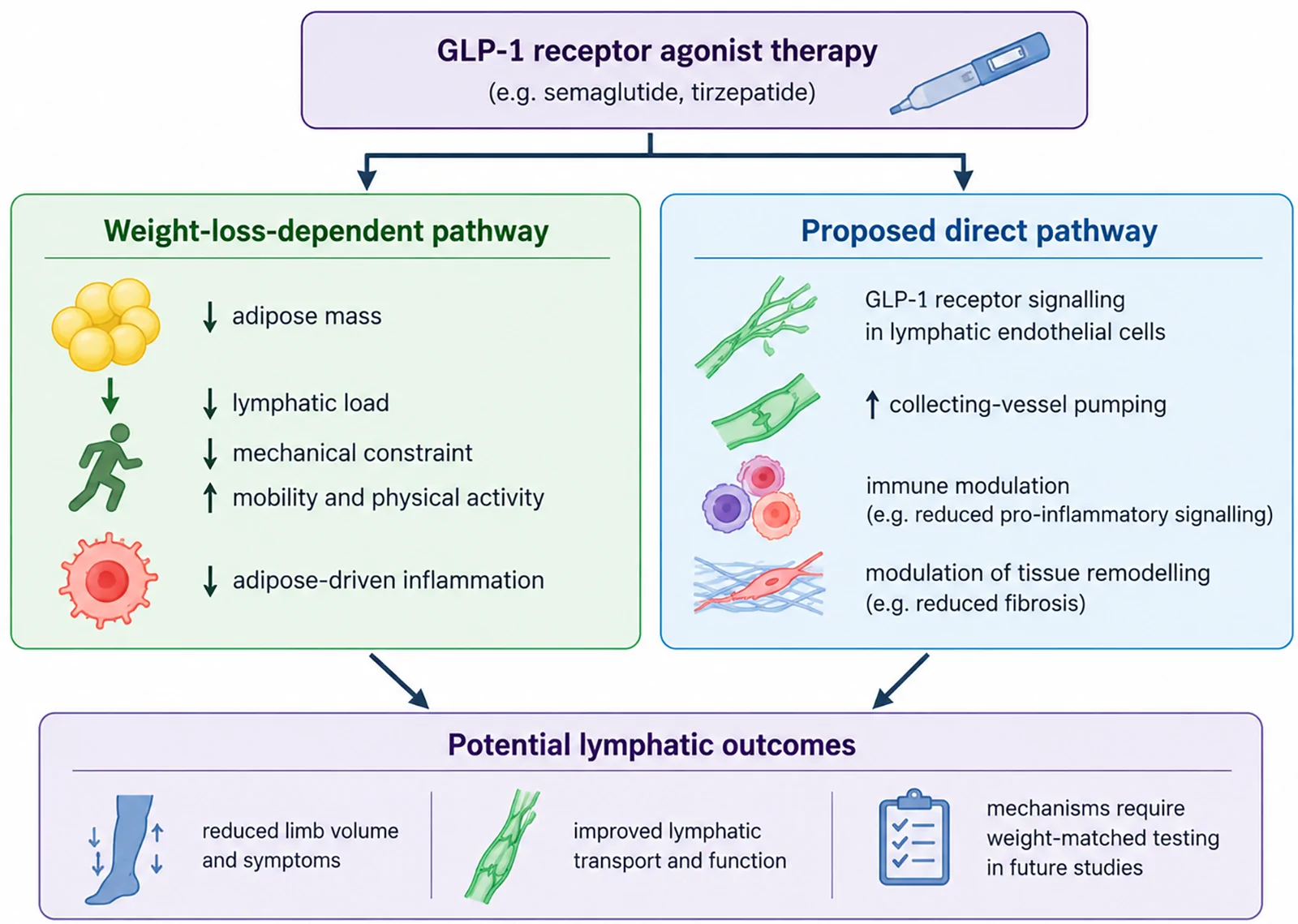
Two-pathway model of GLP-1RA effects on lymphatic function. GLP-1, glucagon-like peptide-1; GLP-1RA, glucagon-like peptide-1 receptor agonist.

Testing these pathways separately makes the proposal strictly falsifiable and yields several key clinical predictions. 1) The unloading prediction, that if systemic unloading is the primary mechanism, lymphatic improvement should strictly parallel the trajectory of fat mass reduction. Furthermore, the lymphatic response should closely resemble outcomes seen in patients achieving comparable weight loss through bariatric surgery or intensive non-pharmacological lifestyle interventions. A fall in limb volume without a measurable improvement in underlying lymphatic transport would support tissue unloading rather than the restoration of vessel function. 2) The direct modulation prediction, that a drug-specific effect on the lymphatic vasculature should manifest independently of significant adipose tissue reduction. This implies that early functional changes, such as increased lymphatic pumping frequency or enhanced tracer clearance on functional imaging, should be detectable before major weight loss is achieved, or remain statistically significant after adjusting for changes in body composition. 3) The phenotype prediction, that because early-stage lymphedema is predominantly a functional failure of vessel contractility, it should be highly responsive to direct GLP-1 receptor modulation. Conversely, advanced lymphedema characterized by profound fibro-adipose remodeling and anatomical disruption may exhibit a blunted response to both pathways (11).

## 7 How should the hypothesis be tested?

Future studies should begin with careful phenotyping. Primary and secondary disease, upper- and lower limb involvement, obesity-induced and cancer-related lymphedema, and lipedema-associated phenotypes should not be pooled indiscriminately. Given that lymphatic vasculature remodels completely differently in SAT versus VAT, patients must be stratified by their baseline fat distribution (11). Duration, stage, prior nodal surgery or radiation, cellulitis history, compression use, and fibro-adipose burden need to be recorded before treatment.

A pragmatic trial could compare standardized lymphedema care plus a GLP-1RA with the same care alone in patients who already meet an approved indication for obesity or diabetes treatment. However, the mechanistically stronger design requires an active comparison group that achieves similar weight loss through other means, such as bariatric surgery or intensive lifestyle intervention. Randomization, blinded image interpretation, and prespecified adjustment for weight and body composition change would then allow a more credible estimate of any drug-specific effect.

The outcome set should reflect both clinical benefit and specific lymphatic physiology. 1) Clinical and functional phenotype comprising limb volume, bioimpedance, symptoms, physical function, quality of life, compression requirements, and cellulitis should be followed. 2) Repeated functional lymphatic imaging, such as Indocyanine-green lymphography, lymphoscintigraphy, or magnetic-resonance lymphangiography, should be used to answer different anatomical and functional questions. These modalities should be selected prospectively and interpreted centrally rather than added as an exploratory afterthought, to evaluate two distinct parameters: intrinsic collecting-vessel pumping frequency and lymphatic barrier integrity (permeability/leakage) (13). 3) Rather than relying on generic inflammatory panels, studies should track specific molecular drivers of lymphatic dysfunction. Targeted assessments should include markers of nitric oxide signaling (such as iNOS-expressing immune cells), specific adipokines (apelin, galectin-1, ANGPTL2), lipid mediators known to induce permeability (LTB4, PGE2, ceramides), and growth factors like VEGFC (11).

Measurements should also be timed to hypothesized mechanisms. An early study visit, conducted before substantial weight loss has occurred, it is important to detect a prompt drug-specific vascular response on lymphatic muscle cell contractility. Intermediate and longer-term follow-ups could can then capture the secondary benefits related to fat loss, reduced physical constraint, and tissue remodeling.

Durability is a separate but equally important question. Weight is commonly regained after semaglutide withdrawal (14). If the lymphatic phenotype strictly follows weight, continued therapy may be required to maintain off-loading. However, the persistence of improved lymphatic pumping or tracer clearance despite partial weight regain would be highly compatible with lasting vascular restoration. Withdrawal studies will therefore be essential, though they should carefully account for concurrent activity, compression adherence, and standard lymphedema care throughout the study period (11). Core design elements for studies intended to distinguish weight-loss effects from direct lymphatic effects are summarized in **Table 3**.

**Table 3:** Core elements of a trial designed to distinguish weight-loss effects from direct lymphatic effects.

Design domain	Recommended approach
Population	Adults with obesity and a well-defined lymphedema phenotype; stratify by fat distribution (visceral vs. subcutaneous predominance), cause, site, stage, duration, and fibrosis
Comparison	Standardized lymphedema care plus GLP-1RA versus standardized care; include an active weight-matched comparator when feasible
Clinical outcomes	Standardized limb volume, symptoms, function, quality of life, compression needs, and cellulitis incidence
Lymphatic outcomes	Repeated ICG lymphography, lymphoscintigraphy, or MRI lymphangiography with blinded central assessment
Mechanistic measures	Body composition, inflammatory and adipose markers, endothelial/vascular measures, and matrix-turnover markers
Timing	Early assessment before major weight loss, intermediate assessment during weight loss, and long-term follow-up including withdrawal where appropriate
Key analysis	Test whether lymphatic improvement exceeds that predicted by weight and body-composition change alone

ICG, indocyanine green; GLP-1RA, glucagon-like peptide-1 receptor agonist; MRI, magnetic resonance imaging..

## 8 Clinical implications and safeguards

GLP-1RAs should not yet be prescribed solely for lymphedema. A more immediate application and evidence-based application is to proactively recognize lymphedema as a relevant, modifiable obesity complication when a patient already qualifies for pharmacological weight management or diabetes treatment. In that setting, systematically recording limb volume, symptoms, compression use, and cellulitis incidence can generate invaluable real-world clinical data without converting observation into an unproven indication.

Current management of lymphedema remains primarily conservative. Complete decongestive therapy (CDT), which combines compression, exercise, skin care, and patient education, is the standard of care (19, 20). Reported outcomes vary with disease etiology, stage, and treatment protocol, although intensive CDT can achieve limb-volume reductions of up to approximately 70% (19, 20). Selected surgical techniques, including lymphovenous anastomosis, vascularized lymph node transfer, and liposuction, may provide additional benefit in appropriately chosen patients (21, 22). However, long-term compression and self-management are often required, and no currently available treatment consistently restores normal lymphatic function across the spectrum of chronic lymphedema.

Established lymphedema care remains necessary. Compression, movement and exercise, skin care, treatment of venous disease, and appropriately selected surgery should continue, as these modalities manage the chronic fibro-adipose tissue remodeling that pharmacological intervention may not fully reverse. Compression garments will frequently need refitting as weight, limb volume and interstitial fluid shift.

Furthermore, the metabolic consequences of rapid weight loss require clinical vigilance. Aggressive nutritional strategies for muscle preservation, prioritizing adequate dietary protein intake and resistance exercise, are critical to offset the loss of lean mass, particularly in older, frail, or sarcopenic patients. Enthusiasm for a novel systemic therapy should not lower the evidentiary threshold for safety (11). Gastrointestinal adverse effects, gallbladder disease, dehydration, cost, and long-term adherence remain relevant.

Looking forward, innovative drug delivery technologies currently in development may address both the systemic side effects of these medications and the uniquely localized nature of lymphedema. Gastrointestinal adverse events, such as vomiting, nausea, and diarrhea, are the most common side effects of current GLP-1 therapies and are largely driven by delayed gastric emptying. To mitigate these effects and overcome the compliance barriers associated with chronic injections, researchers are developing continuous-acting implantable pumps and smart-acting electronic devices capable of fine-tuning systemic GLP-1 release. Perhaps most relevant to massive localized lymphedema, advanced targeted delivery systems, such as injectable hydrogels and transdermal microneedle patches, are being designed to transport active compounds directly to specific localized adipose tissues. These highly localized delivery methods could theoretically target the specific fibro-adipose burden of a lymphedematous limb directly, potentially maximizing lymphatic endothelial recovery while minimizing the systemic gastrointestinal side effects of GLP-1 therapies (15).

Conversely, the failure of lymphedema to clinically improve is not a valid reason to withdraw a treatment that is actively delivering established metabolic or cardiovascular benefits. The primary metabolic indication and the secondary lymphatic research question should remain formally separate in clinical practice.

## 9 Conclusion

The management of chronic lymphedema remains challenging, and the introduction of disease-modifying systemic therapies would represent a major paradigm shift. Current evidence, however, does not yet establish GLP-1RAs as a targeted treatment for human lymphedema.

The proven effects of these agents, substantial adipose tissue loss and improved metabolic health, may alleviate the mechanical constraint, interstitial fluid load, and adipose-driven inflammation that perpetuate lymphatic stasis. Alongside this weight-loss-dependent unloading, a direct pharmacological effect on the lymphatic vasculature is a highly compelling hypothesis. Experimental data confirming high natural GLP-1 penetrance in mesenteric lymph, enriched receptor expression on collecting lymphatic vessels, and NO-mediated restoration of lymphatic pumping capacity provide a robust biological rationale for direct modulation.

However, translating this physiological signal into therapeutic proof requires rigorous, prospective controlled studies. Future trials should distinguish between systemic unloading and targeted lymphatic modulation by combining precise clinical phenotyping, continuous functional lymphatic imaging, and active weight-matched comparison groups. Additionally, as novel multi-agonists and localized delivery technologies enter the clinic, they may offer even more precise tools to target fibro-adipose accumulation. Until such evidence matures, GLP-1RAs should be utilized for approved metabolic indications alongside established lymphedema care, with any lymphatic benefit regarded as a promising, testable clinical signal rather than a definitive treatment claim.

## Data Availability

The original contributions presented in the study are included in the article/Supplementary Material. Further inquiries can be directed to the corresponding authors.
